# Performance investigation of different headlights used in vehicles under foggy conditions

**DOI:** 10.1038/s41598-023-31883-3

**Published:** 2023-03-22

**Authors:** Taylan Sefer, Ramazan Ayaz, Ali Ajder, Ismail Nakir

**Affiliations:** 1 Department of Electrical Engineering, Yildiz Technical University, Esenler, 34220 Istanbul, Turkey; 2 Clean Energy Technologies Institute, Yildiz Technical University, Istanbul, Turkey

**Keywords:** Electrical and electronic engineering, Engineering, Optics and photonics

## Abstract

In this study, an experimental study based on the target visibility criteria is carried out for dark and foggy environments, where only vehicle headlights are active. More specifically, the performances of the headlights containing halogen, xenon, and LED light sources in dark and foggy weather conditions are examined and the performance of each headlight is evaluated separately. According to the general result of the visibility experiments, the participants perceived the target object in a shorter time when halogen headlights are used. It is also observed that the average target perceived times of the participant under foggy conditions are at different levels depending on the type of headlights with different correlated color temperature (CCT) and color rendering (CRI) indexes. According to the experimental results performed under foggy conditions, the target detection times of the participants are the shortest at the halogen headlight, the second at the xenon headlight, and the third under the LED lights. It has also been observed that this sequencing is gender-independent. However, the ratio of the detection time of female participants to that of male participants varies between 1.72 and 3.65 times. In other words, female participants perceive the targets in a much longer period than male participants.

## Introduction

Light consists of photons is a special form of radiant energy that affects the human eye and travels in the form of tiny particles and electromagnetic waves, which causes a visual perception in the observer's eye. The structure of a human eye consists of a tough spherical layer, vascular layer, and inner layer i.e. retina. The retina parts consist of cone and rod cells, which are sensitive to light and colour. Rod cells have equal spectral sensitivity and provide vision at low light levels (scotopic vision). Cones are active at higher light levels (photopic vision) and allow the perception of colour. Mesopic (mixed) vision occurs when both cones and rod cells work together at low and medium light levels^1–4^.

Light hitting a surface can be reflected, transmitted, or absorbed depending on the nature of the surface. The application of the light to objects and/or to their surroundings is called lighting. Lighting applications such as home, school, industrial facilities, and highways are examples of the main lighting applications. As one of the most important of these, road lighting not only provides safe and comfortable driving and but also ensures order and safety for pedestrians^2^. Especially successful road lighting applied by the standards improves the visual conditions of drivers and pedestrians and reduces night accidents^5–10^.

The safety and comfort of road users reduce significantly on poorly illuminated roads. Hence, it is inevitable to have well-designed road lighting to get better visual performance. One way of reducing the risk of accidents at night times is to improve the lighting conditions during driving^11^. The driver is either drowsy or asleep in most night-time accidents^12^. The main purpose of roadway lighting is to provide visual performance and comfort for the driver and also to keep the driver alert^8,12^. Compared to daytime accidents, traffic accidents occurring at night times are more frequent and more severe^5,13^. Some of these accidents may be caused by vision problems. Studies conducted by many institutions and organizations in different countries show that good road lighting can help to reduce night accidents by 30%^9,12,14–19^. Roads with an average luminance level of 1.2–2 cd/m^2^ on the surface have 20–30% lower accident rates than road surfaces with 0.3–1.2 cd/m^2^. This shows us that visual performance directly affects traffic and roadway safety. Road lighting also serves to deter violence, vandalism, and crime^12^. In addition, human visual perception varies greatly in the mesopic region according to the spectral power distribution of light sources. Some studies show that shorter wavelength light sources lead to brightness perception and luminous efficiency^20–23^.

Road lighting has been realized by using a wide variety of light sources for decades. However, on roads where there is no lighting system installed, drivers have to use the headlights of the vehicle only for their visual needs. These headlights, which are a great need for drivers and traffic safety, have been becoming more technological and more efficient every day. The purpose of the front and rear lighting systems of the vehicle is completely different from each other. For this reason, different materials and operating needs are available for the front and rear lighting systems. The main function of the front lighting system is to illuminate the road. However, the purpose of the rear lighting system is to notify different actions such as brake, stop and signal to become more noticeable to other drivers. Nowadays, light sources such as halogen, high-intensity discharge (HID), and LED lamps are used in vehicle headlight systems.

It is a well-known fact that traffic accidents occurring on vehicle roads are considerably higher than that of other transport systems. It has been stated on the World Health Organization (WHO) Global Status Road Safety Report published in 2008 that deaths due to road traffic accidents are 1.35 million per year and road traffic accidents are the eighth leading cause of death globally^24^**.**

Various meteorological events such as rain, snow, and fog reduce the visibility range of drivers. Even, in some cases, the drivers may be faced with situations where the visibility is almost zero^5^. Among these meteorological events, fog seriously disturbs the drivers and gives the drivers a hard time while driving. For this reason, very dangerous accidents occur, resulting in death and injuries. Since the foggy weather conditions reduce the vision of vehicle drivers, the risk of traffic accidents also increases, especially at nightfall times. For example in Turkey, traffic accident existing in rainy weather conditions has the highest rate, while the second-highest accident rate happens in foggy weather conditions^25^. Fog is a type of cloud close to the ground and can be categorized as either warm or cold fog depending on whether it consists of water droplets or ice crystals. A fog event can reduce visibility to less than 1000 m^26^. Therefore, the transmission of light is reduced in foggy conditions as these droplets capture or scatter light, which results in lower visibility for drivers. In addition, some of the light is lost or a white wall effect occurs due to the reflected light^6^.

Yukio Akashi et al. conducted a field study along a street illuminated by metal halide or high-pressure sodium light sources. The driver was asked to perceive the direction of the off-axis target approaching or moving away from the street and accelerate or brake accordingly. At the same photopic light level, off-axis perception times were found to be shorter under a white metal halide light source. They also proved that the perception times of both braking and acceleration decrease as the combined luminance increases^27^.

The lighting performances of two LED sources at 3100 K and 6500 K colour temperatures were evaluated in day and night fog conditions. It has been observed that the object recognition rate of LEDs with low colour temperature is higher than LEDs with high colour temperature^28^. Dong et al. examined 6 different LED light sources (3500, 4000, 4500, 5000, 5700, and 6500 K) to evaluate the effect of correlated colour temperature (CCT) on the visual performance of LEDs under mesopic conditions^29^. The results showed that while the mesopic luminance increased wıth increase of CCT and the perception times of the observers in mesopic vision decreased^29^. In another study, the fog penetration ability of tungsten filament lamp (TFL), LED, metal halide (MH), and high-pressure sodium vapour lamp (HPS) in mesopic vision was investigated. It has been determined that the ability of TFL to penetrate the fog is the best, while the LED is the worst. It has been determined that yellow light has a better ability to penetrate the fog than white light and therefore LEDs wıth low colour temperature would be more suitable for street lighting^30^. However, in another similar study, a new luminance calculation method was identified, which explains the mesopic vision and its ability to penetrate fog. In another study, 6 LED light sources with different colour temperature values varying between 3500 and 6000 K were also investigated. It was calculated for low transmittance rates that LED colour temperature decreases as the increase in luminance. For high transmittance rates, it was also determined that LED lamps with high colour temperature would be the most suitable for street lighting^31^.

Steve Fotios et al. studied the effect of fog on the detection of driving hazards under dark weather conditions^32^. An experimental study to detect hazards in the peripheral field of vision was conducted for a vehicle that is changing the lane on a road surface having obstacles. It ıs stated that the effect of luminance, S/P ratio, and fog densities was also investigated. It has been proven that increased luminance and a decrease from thick to thin fog cause a decrease in detection speed and reaction time^32^. In an experimental study, the peripheral perception performance of drivers during the transition between illuminated and unilluminated road sections was investigated. Tests were conducted between a luminance of 0.1 cd/m^2^, 1.0 cd/m^2^ and 2.0 cd/m^2^. While the luminance increase from 0.1 cd/m^2^ to 1.0 cd/m^2^ improved detection, 2.0 cd/m^2^ did not improve the luminance detection. The 0.65 and 1.40 S/P ratios were examined at 1.0 cd/m^2^ luminance, and it was found that it does not affect detection performance. As a result, it was determined that the visual performance increased and reached a stable point under 1.0 cd/m^2^ luminance^33^. In another experimental study, the illuminance level and luminance attenuation measurements were conducted under varying fog density. It has been observed that shorter wavelengths (blue and green) are 1.5 times less scattered than longer wavelengths (yellow-green, amber/ochre, and red). In addition, it was also observed that the colour temperature of the white LEDs increased with the increase in fog density depending on the distribution of light in wavelength^34^.

This study includes an experimental study based on the visibility criteria in dark weather conditions where only vehicle headlights are active and road lighting is not available. The performances of various technologies including halogen, HID (xenon), and LEDs used in vehicle headlights have been investigated in a dark environment and foggy weather conditions. In the experimental study, the performance of each headlight was evaluated separately. The aspects listed below in this study are different from other studies in the literature:

- Up-to-date technologies halogen, LED, and xenon light source-based headlights are used in the experimental study.
- Light transmittance ratios of all spectra at equal illuminance levels are measured and the relationship between them is observed.
- The visibility of yellow and red-coloured targets under fog conditions is tried to be determined by measuring the object detection times of female and male participants of various ages. These measurements are made for real headlights containing halogen, LED, and xenon technologies separately.

## Experimental study

The experiment was approved by The Ethical Committee of Yildiz Technical University (2022.09) and carried out according to the Declaration of Helsinki. All methods in the study were carried out in accordance with relevant guidelines and regulations. Each subject was kept informed of the research aim and all the details of the study prior to starting the experiment. Informed consent was obtained from all subjects.

The performances of halogen, LED, and HID (Xenon) light sources having different spectrums most used in the headlights of motor vehicles have been investigated under foggy and dark weather conditions. First of all, the transmittance levels of these light sources under fog are measured. The visibility of the object under these respective light spectrums (only headlight sources are active and there is no road lighting) is then tested under foggy and dark weather conditions for different participants.

### Headlights and measurement setup

Light sources with 3 different technologies are generally used in automotive headlights^35,36^. For this reason, halogen, LED, and xenon fog lights are preferred in this study. The transmittance of halogen, LED and xenon light sources with different spectrums under fog have been measured for the headlights detailed below:

- The halogen light source uses in Toyota Corolla model vehicles is selected as the halogen light source. This light source operates with a voltage of 12 V and has a power of 55 W. Its brand is Philips and it is a HIR2 type lamp with catalog number 9012.
- LED-based headlights used in Toyota CH-R model vehicles are selected as the LED light source. This light source operates with a voltage of 12 V and has a power of 8 W.
- As the xenon light source, the xenon-based headlights used in Ford Transit vehicles are selected. This light source operates with a voltage of 12 V and has a power of 25 W. Its brand is Philips and it is a D5S type lamp.

**Figure 1:**
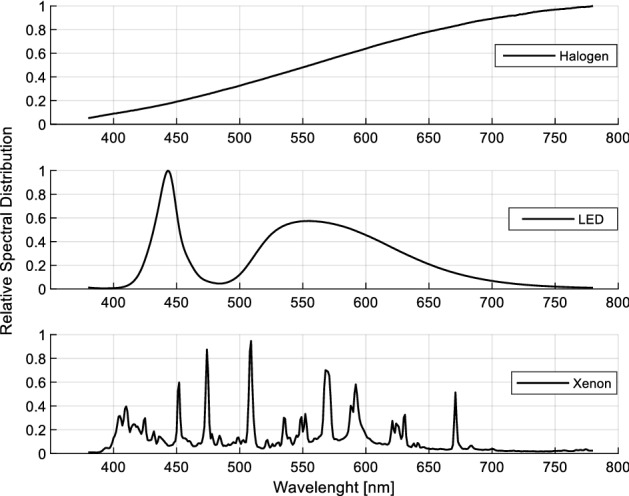
Relative spectral power distributions of halogen, LED, and xenon light sources.

Relative spectral distributions, CCT, and CRI values of light sources are measured in the integrating sphere and a high-accuracy array spectroradiometer (Everfine HAAS-2000), and these values are given in Fig. 1 and Table 1, respectively.

**Table 1:** Colour characteristics of the headlights used in the measurements.

Headlight Type	CCT (K)	CRI
Halogen Headlight	3031 K	99.8
LED Headlight	5122 K	65.7
Xenon Headlight	5098 K	72.2

While the halogen headlight has a color temperature of 3031 K, LED and xenon headlights have 5122 K and 5098 K, respectively.

Measurements are carried out in Yıldız Technical University Lighting Laboratory. The measurement setup and experimental conditions are listed below. In the measurements, a 100 × 80 × 100 cm box, the inner surface of which painted in matt black, is used.

- Halogen, LED and xenon light sources are placed on a wooden base between the participant and the box where the targets are placed. Thus, the users are given the impression of driving.
- The position of the headlights does not obstruct the view of the participants.
- The headlights and the experimental area are covered with matte black curtains along a corridor on the right and left sides to avoid glare for the participants.
- A diffuser is placed in front of the light sources (towards the target direction) to ensure that the light is distributed evenly and equally.
- Where the target is placed, an equal illumination level is provided under all light sources.
- Everfine Z2000 model multi-light meter is used in the measurements of the illuminance.
- Antari Z800II fog machine which generates 1.416 m^3^ fog per second, and the medium one of three types of fog liquid (light-medium-heavy) density is used for the production of fog.
- It is provided that the fog is given to the box from a point on the back of the box where the targets are placed.
- The front part of the box where the target is placed is covered with thick cardboard, the top half of which is painted black, and its half of the bottom is glass. The upper part of the glass here is also covered with matte black fabric to avoid glare when the headlights are activated.
- The perception times of the participants are measured with a counter.

**Figure 2:**
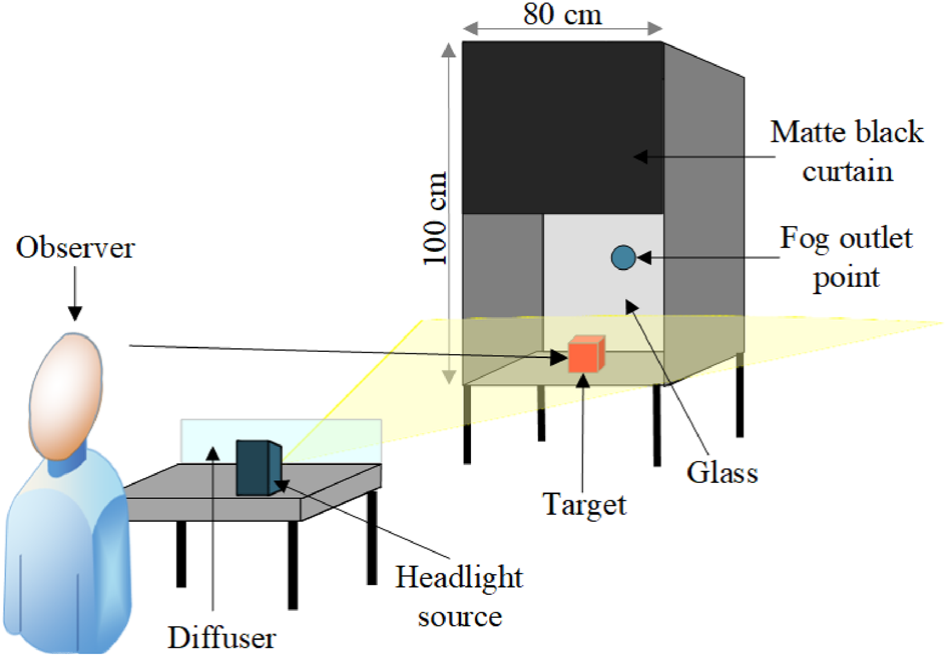
The measurement set up.

The measurement setup described above is illustrated in Fig. 2.

**Figure 3:**
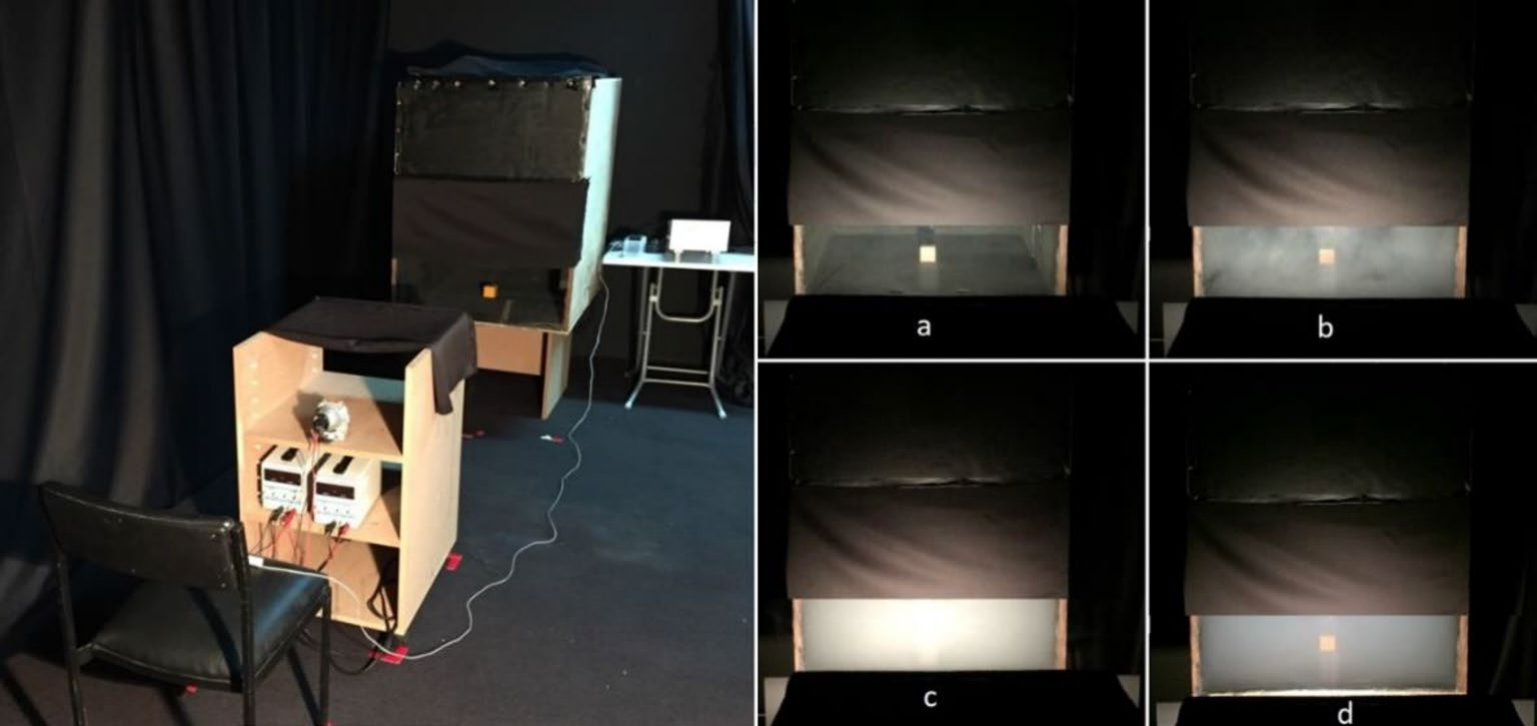
Experimental measurement setup (left side); the participant's field of view at different times during the experiment (right side): (**a**) no fog, (**b**) the first moment of the fog first entrance, (**c**) homogeneous distribution of the fog, (**d**) the fog decreases over time.

The visuals of the measurement setup and target area at different moments are given in Fig. 3. Figure 3a and b show the fog-free condition, and the first moment of fog entry, respectively. In both cases, the measurement setup is not shown to the participants. Figure 3c and d present the case of homogeneous distribution of the fog and the situation in which the target is completely seen after detection, respectively.

### Fog density determination

To determine the density of the fog, illuminance level (lux) values are measured in a vertical direction at the point where the target object is located. Lux level results are obtained under the same reference light source. First, the vertical illumination level is measured without fogging inside the box. This illuminance is given in the last column in the Table 2 as fog free conditions for each measurement. Then, an equal amount of fog (approximately 12.744 m^3^ for 9 s) is introduced into the box from the same point as the fog machine. After the fog is emitted into the box, the illumination level is measured and recorded at the same points for certain periods. Measurements are repeated three times under the same conditions to verify that the fog density inside the box is the same each time. Approximately the same results are obtained for each measurement and the results are given in Table 2.

**Table 2:** Measurement results to verify the fog density.

	Time (min)									Fog free conditions
	0.5	1	3	5	7	9	11	13	15	
Measurement-1 [lx]	49	52	68	84	95	108	116	123	129	247
Measurement-2 [lx]	50	54	71	86	98	109	117	125	130	247
Measurement-3 [lx]	50	53	71	87	97	107	116	123	130	247

As can be understood from Table 2, the amount of change of fog over time remains the same every time as long as the amount of fog given to the box is the same. In other words, the fog layer (density) between the participant and the target shows the same change over time. Thus, it is ensured that the fog density inside the box is the same for each experiment.

### The measurement method

Before the experiment, vertical illuminance levels at the same height as the target objects are measured in front of each headlight light source. Illuminance level values are taken from different points on the same horizontal axis in the box. The luminous fluxes of the headlights are adjusted so that the illuminance levels measured at each point for each headlight are equal. The measured illuminance levels are almost equal at each point and vary by ± 5 lx. Surface luminance value shows the same characteristic for observer angles between 20° and 45°^37^. For this reason, the observer angle has been adjusted to 20° and the target viewing angle to 1°. During the experiment, the laboratory temperature is kept constant at 24 ± 1 °C and the humidity at 60%.

Measurements are taken for a total of 12 participants, 6 males and 6 females, aged between 21 and 36 years. Participants had no prior idea about the measurements. Before starting the experiments, Ishihara colour blindness test is applied to all participants, and it is determined that none of them are colour blind. Only two of the participants, one male, and one female, wear glasses and have normal vision. No discrepancy is observed in the results of participants wearing glasses.

**Figure 4:**
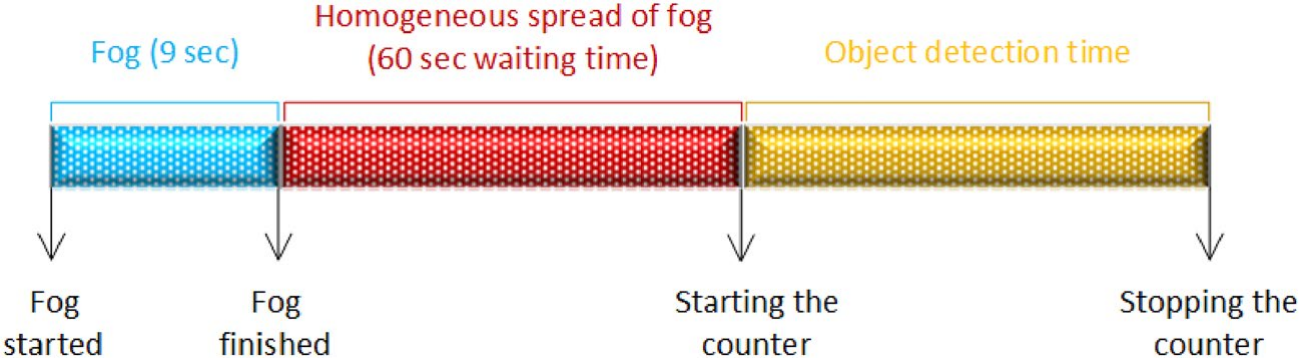
The measurement process.

After the headlights are turned on and stabilized, the participants are allowed to look at the box for 5–10 min to adapt to the headlights. For each measurement, the location of the target is changed, and participants are prevented from seeing it. Objects are randomly positioned in the box on the horizontal axis for each measurement. In addition, the fogger is integrated with the counter button, so that fog emission is provided inside the fogger box for only 9 s (12.744 m^3^). It has waited for 60 s (including the first 9 s) to distribute the fog evenly and homogeneously inside the box. Participants are asked to imagine driving in the dark and in foggy weather. At the 61st second, the black curtain placed between the participant and the box is taken away. As soon as the participants perceive the target, they stop the counting process by pressing the button in their hands. In this way, the performance of the headlights containing different light sources under fog is measured over the perception times of the participants. Each experiment is repeated at least twice for each participant to be sure of the experiment results. The entire procedure described above is repeated for each experiment. The measurement process is given in Fig. 4.

### Relative luminance of targets

In the measurements, small yellow and red cubes of 5 × 5 × 5 cm are used as targets. These colours are chosen in this study because the vehicle rear lights are red (stop lamp) and yellow (turn signal) in traffic. First, the light sources are powered up and waited for it to become stable. The illuminance levels created by all light sources used in the measurements are equalized at the same distance and on the same surface.

**Figure 5:**
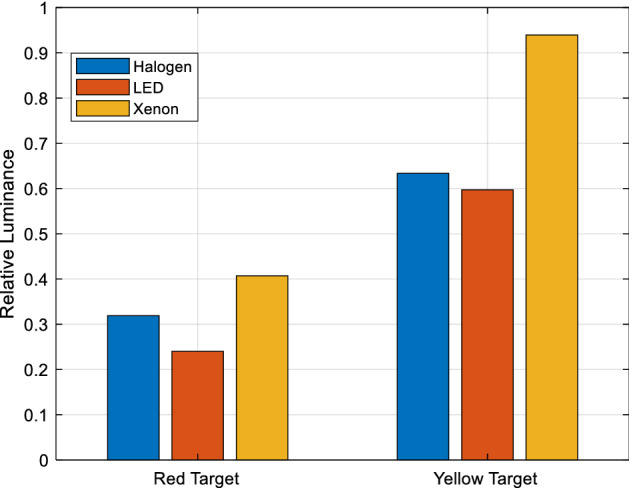
Relative luminance of target objects.

The luminance camera (TechnoTeam LMK 98-4) is positioned at a 20° measurement angle and focused on the white barium sulphate surface placed in the centre of the box. First, the luminance of the reference material and then the luminance of yellow and red targets are measured under the same light source. For three different headlights (halogen, LED, xenon), the luminance of the yellow and red targets are compared separately with the luminance of the reference material to obtain relative luminance. Relative luminances are given in Fig. 5.

As for relative luminance, the relative luminance of the red target under different spectrums are close to each other, but the relative luminance values from the biggest one to the lowest one are measured as xenon, halogen, and LED, respectively. In the relative luminance of the yellow target under different spectrums, the relative luminance of the halogen and LED spectra are found to be very close to each other. It is seen that the relative luminance of the xenon spectrum is higher than the others. It is also observed that the relative luminance of the xenon spectrum in both colours was higher than the other spectra. Also, under all spectra, it is seen that the relative luminance of the yellow target are much higher than the relative luminance of the red target.

## Measurement results

### Headlight transmittance rates under fog

**Figure 6:**
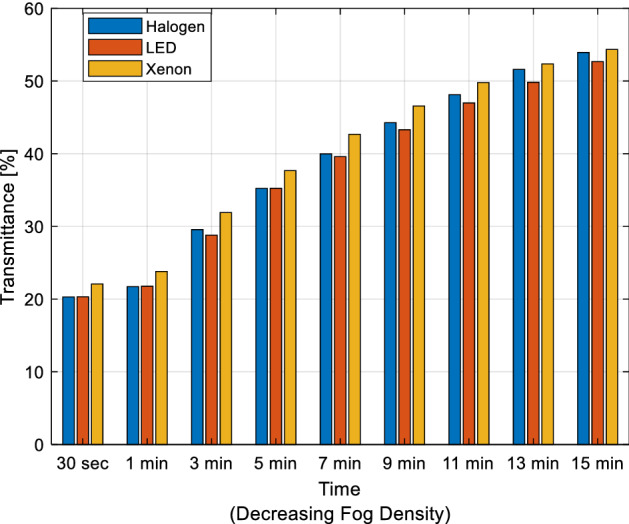
The transmittance of headlight light sources.

As with fog density measurement, the fog is given into the box for 9 s for each light source. The fog is expected to distribute homogeneously inside the box for 30 s. Then, a total of 9 illuminance levels are measured at the 30th second and 1, 3, 5, 7, 9, 11, 13, 15 min. As a result, transmittance ratio changes are obtained for the decreasing fog density over time. Each illuminance value is calculated in percent by proportioning the value of the illuminance level in a fog-free environment. The transmittance ratio values of vehicle headlights with different technologies in medium fog density are determined with the measurements taken. The transmittance of headlight light sources depending on the decreasing fog density is given in Fig. 6.

In varying fog density, the transmittance values under xenon headlights are approximately 2–3% higher than halogen and LED headlights in almost every measurement. The transmittance ratio of halogen headlights is nearly 0.5–1% higher than LED headlights at each measurement. In other words, the transmittance ratios of these two headlights are approximately equal to each other. It is understood that the transmittance is around 20% in the 30th second for all three headlights, and these rates increase as the fog density decreases over time and reach the level of 53–54% at the end of the 15th minute and almost equalize.

Since transmittance is insufficient alone to evaluate vehicle headlights’ performance under fog conditions, the luminance of the target objects should also be considered. However, the luminance camera measures the luminance of fog instead of the target's luminance due to the fog in front of the object in the luminance measurements. Therefore, user-based tests that consider color temperature and luminance parameters should be conducted for a more realistic performance evaluation in foggy conditions.

### Participant based measurement results

According to the measurement method described above, measurements are made with different targets under different headlights for each participant. The analysis is separately carried out with yellow and red target objects to reveal the performance of the headlights by recording the perception times of the participants. Although the participants press the button when they see the yellow or red target objects, their detection times vary due to the different sensitivity of the participants compare to the other participants. To interpret the results better, the following calculation method has been considered.

The method is used to analyze the detection times measured for yellow or red target objects under different headlight light sources. The shortest detection time has been subtracted from the longest detection time, 30% of the difference is calculated and the value found is added to the shortest detection time. This value is accepted as the reference detection time. The detection times from the shortest detection time to reference are accepted as short-time detection, others as long-time detection. This expression is given in Eq. (1).

1 $${DT}_{ref}= \frac{\left({DT}_{l} - {DT}_{s} \right)*30}{100}+{DT}_{s}$$

where, *DT*_*ref*_ , reference detection time; *DT*_*l*_, longest detection time; *DT*_*s*_, shortest detection time; [*DTs−DT*_*ref*_] interval, short time detection; [*DT*_*ref*_*−DT*_*l*_] interval, Long time detection.

**Figure 7:**
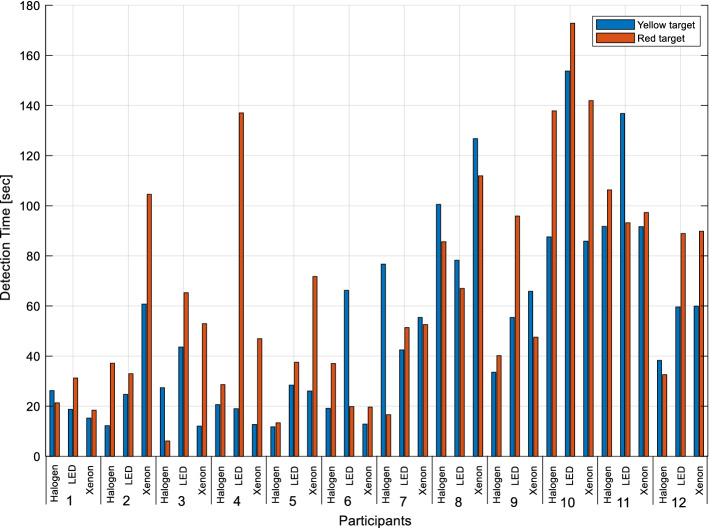
Detection times of target objects under each headlight (Participants 1–6 male, 7–12 female participants).

Detection times of all participants for yellow and red target objects under halogen, LED and xenon headlights are given in Fig. 7.

**Figure 8:**
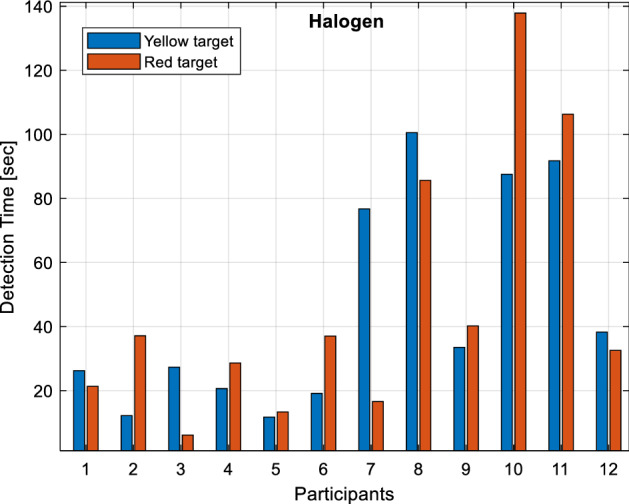
Detection times under the halogen light source.

Detection times of yellow and red targets under halogen headlights are given in Fig. 8. As can be seen from the figure, participants detect the yellow target in the range of 11.75 to 100.55 s. and the red target in the range of 6.15 to 137.86 s. It is observed that 66% (8 people) of the participants perceive the yellow target object, and 75% (9 people) perceive the red target object below the reference detection time.

**Figure 9:**
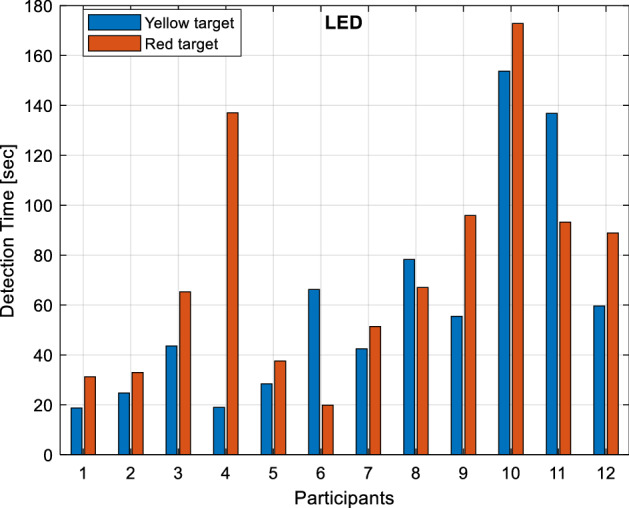
Detection times under the LED light source.

The detection times of yellow and red target objects under LED light sources are given in Fig. 9. As can be seen from the figure, participants detect the yellow target in the range of 19 to 153.75 s. and the red target in the range of 19.85 to 172.85 s. It is observed that 58% (7 people) of the participants perceive the yellow target object, and 50% (6 people) perceive the red target object below the reference detection time.

**Figure 10:**
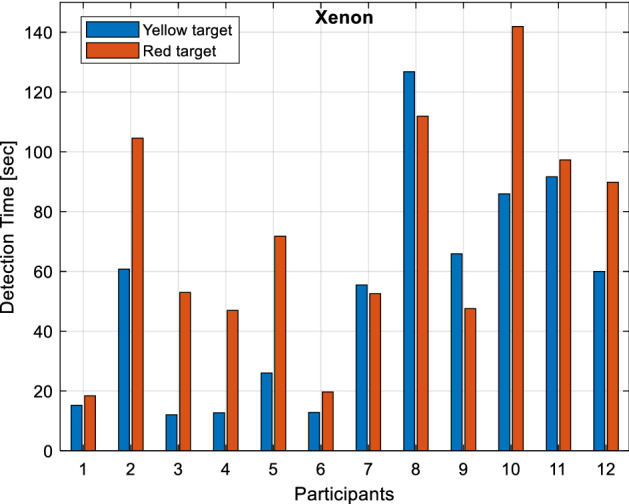
Detection times under xenon light source.

Detection times of yellow and red target objects under the xenon light source are given in Fig. 10. As can be seen from the figure, participants detect the yellow target in the range of 12.05–126.8 s. and the red target in the range of 18.4–141.93 s. It is observed that 42% (5 people) of the participants perceive the yellow target object, and 50% (6 people) perceive the red target object below the reference detection time.

The same participants can see both colors (yellow and red) below the reference detection time. Percentages are calculated for each color separately. Therefore, the number of participants perceiving the target objects below the reference detection time varies under different light sources.

**Figure 11:**
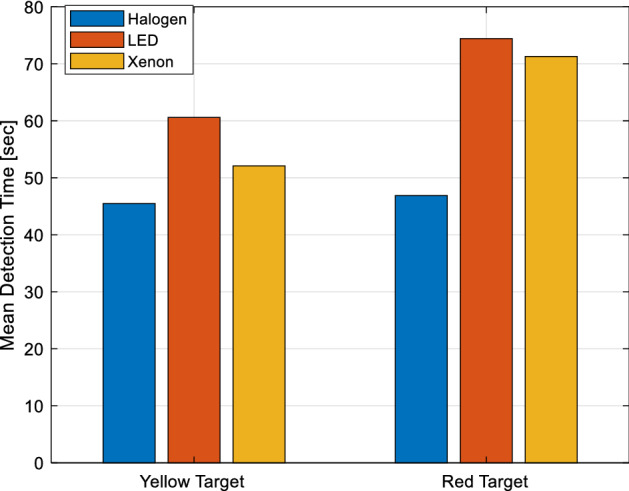
Average detection times of all participants for yellow and red targets.

Detection times of all participants are averaged for yellow and red target objects under different spectra and are given in Fig. 11. These average values are given for yellow and red targets comparatively. These average values are found to be almost equal under halogen headlights. More specifically, the ratio of red target detection time to yellow target detection time is 1.03. Under the LED light source, the yellow target is detected in a shorter time and the ratio of the detection time of the red target to that of the yellow target is 1.22. Under the xenon light source, the yellow target is detected in a shorter time as well. The ratio of the detection time of the red target to that of the yellow target is 1.37 in this case. In general, the detection times of both yellow and red targets are shorter under halogen headlights than under other headlights.

**Figure 12:**
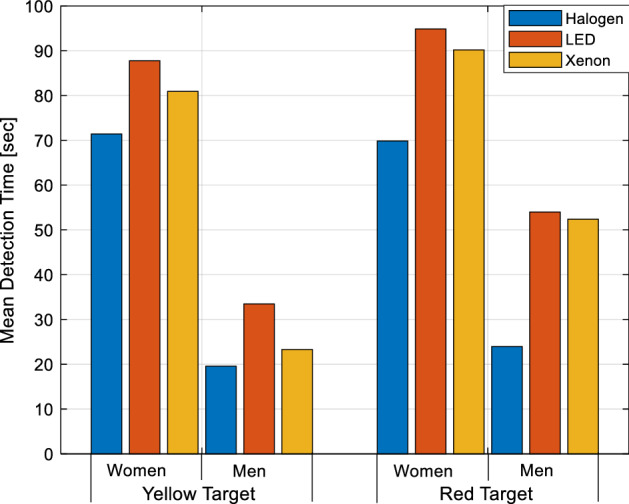
Average detection times of female and male participants.

Separate results for the averaged detection times for female and male participants are given in Fig. 12. It is observed that the detection time of male participants for both targets is shorter than that of females.

Detection times of female and male participants are also evaluated in separate groups. Accordingly, female participants’ detection times of yellow and red targets under halogen, LED and xenon headlights are very close to each other. However, this relative difference as shown in Fig. 12 can be as high as 1.22, 1.61, and 2.25 for male participants.

Under the halogen headlight, the detection time for male participants is 3.65 times less for the yellow target and 2.91 times for the red target than for the female participants. The detection time of male participants under LED headlights is 2.62 times less for the yellow target and 1.75 times for the red target than for the female participants. Under the xenon headlight, it is seen that the detection time of male participants is 3.47 times less for the yellow target and 1.72 times for the red target than for the female participants. As a result, it can be said that the perception times of men for both colors in all spectrums are shorter than that of women. According to another general result, it is determined that both female and male participants have shorter detection times for the target of both colors respectively for halogen, xenon, and LED headlights. In the study of Jain et al., reaction times to visual stimuli are slower in women compared to men^38^. Therefore, the comparison of detection times obtained in this study is similar to their observation.

## Conclusions

In this study, the performances of halogen, LED and xenon light source-based vehicle headlights under fog are investigated for dark weather conditions. The performances of these headlights with different spectra under fog are measured based on two main parameters. The parameters are the transmittance levels and the participants’ detection times for the yellow and red targets (under the fog and dark weather conditions).

Based on the previous studies in the literature, results are obtained only in mesopic conditions based on CCT measurement. Although personal tests are performed, only one light source has been examined. Examinations are made by measuring the light transmittance of different light sources in the fog, without making individual-based tests. Object recognition analysis is carried out in day and night fog conditions.

In this study, different issues from the studies in the literature are considered. More specifically, halogen, LED, and xenon light-based real headlight modules commonly used in existing motor vehicles were tested. Light transmittance is measured at equal illuminance levels in foggy weather conditions, and the results are analysed. However, it has been shown that transmittance measurements made under fog are not sufficient criteria alone to evaluate the performance of vehicle headlights. The human factor is also considered for a more accurate performance evaluation. In this framework, individual tests are conducted for the visibility of yellow and red targets under foggy weather conditions.

For yellow and red targets, the relative luminance value of xenon headlights is found to be higher than other headlights. As for the transmittance measurements, it is observed that the transmittance of xenon headlights was 2–3% higher than the other headlights. However, in user-based experiments conduct at equal illuminance levels, it is seen that detection times under halogen headlights are lower than detection times under xenon and LED headlights. This is because of the white wall effect in the eyes of the participants as a result of the white light reflection in a foggy environment. As known, the white wall effect has a negative effect on the human eye and visibility. In addition, it is observed that women perceive both targets later in all spectrums than men in foggy environments in this study.

As a result, conventional halogen light-based headlights are more successful in foggy environments, despite the economical, efficient, and aesthetically pleasing xenon and LED-based headlights. It is thought that drivers with halogen headlights will have better vision conditions in areas where fog is common. For this reason, they will be able to drive more comfortably and safer than other drivers. In addition, it is thought that traffic accidents occurring in foggy areas will decrease if the drivers who drive in these regions prefer halogen lights. R&D activities for light sources and headlight designs are constantly ongoing and performance improvements can be achieved frequently. For this reason, vehicle headlights with a wide range of technological features are still being produced. Therefore, it is necessary to continue such experimental studies and research involving more observers and new headlights. In addition, the use of LED light sources with low correlated colour temperatures may give more successful results, as with halogen light sources. Therefore, these measurements can be performed using LEDs with low CCT.

## Data Availability

The datasets generated during the current study are available from the corresponding author on reasonable request.
